# Additional single dose GnRH agonist during luteal phase support may improve live birth rate in GnRHa-HRT frozen–thawed embryo transfer cycle: a retrospective cohort study

**DOI:** 10.1186/s12884-023-05491-y

**Published:** 2023-03-14

**Authors:** Wei-Shan Chang, Pei-Hsuan Lin, Chia-Jung Li, Chyi-Uei Chern, Yu-Chen Chen, Li-Te Lin, Kuan-Hao Tsui

**Affiliations:** 1 Department of Obstetrics and Gynecology, Kaohsiung Veterans General Hospital, No.386, Dazhong 1st Rd., Zuoying Dist, Kaohsiung City, 81362 Taiwan; 2 Department of Obstetrics and Gynecology, Kaohsiung Armed Forces General Hospital, Kaohsiung City, Taiwan; 3 Institute of Biopharmaceutical Sciences, National Sun Yat-sen University, Kaohsiung City, Taiwan; 4 Department of Obstetrics and Gynecology, School of Medicine, National Yang-Ming University, Taipei City, Taiwan; 5 Department of Biological Science, National Sun Yat-sen University, Kaohsiung City, Taiwan

**Keywords:** Luteal GnRH agonist, In vitro fertilization, Frozen embryo transfer, Hormone replacement therapy cycles, Artificial cycles, GnRH agonist pretreatment

## Abstract

**Background:**

GnRH agonist (GnRHa) has been reported to have direct effects and functional roles in the endometrium and embryos. Several meta-analyses have shown that GnRHa administration in the luteal phase improved the live birth rate or pregnancy rate in both fresh and frozen embryo transfer (FET) cycles. The aim of this study was to investigate whether luteal GnRHa administration could also improve in vitro fertilization (IVF) outcomes in patients undergoing hormone replacement therapy (HRT) cycles with GnRHa suppression.

**Methods:**

The retrospective cohort study included a total of 350 patients undergoing GnRHa-HRT FET cycles. The study group included 179 patients receiving an additional single dose of GnRHa in the luteal phase following embryo transfer. A total of 171 patients in the control group did not receive luteal GnRHa. The baseline and cycle characteristics and reproductive outcomes were compared between the two groups.

**Results:**

Baseline and cycle characteristics were similar between the two groups, except lower AMH levels were found in the luteal GnRHa group than in the control group. The luteal GnRHa group had a significantly higher ongoing pregnancy rate and live birth rate than the control group. The multivariate analysis revealed that luteal GnRHa administration was positively associated with ongoing pregnancy (OR 2.04, 95% CI 1.20–3.47, *P* = 0.008) and live birth (OR 2.03, 95% CI 1.20–3.45, *P* = 0.009). When the subgroup of patients with recurrent implantation failure was analyzed, the multivariate analysis also showed that luteal GnRHa administration had beneficial effects on ongoing pregnancy (OR 4.55, 95% CI 1.69–12.30, *P* = 0.003) and live birth (OR 4.30, 95% CI 1.59–11.65, *P* = 0.004).

**Conclusions:**

Our data suggest that the addition of one luteal dose of GnRHa may improve the live birth rate in patients undergoing the GnRHa-HRT protocol.

**Supplementary Information:**

The online version contains supplementary material available at 10.1186/s12884-023-05491-y.

## Introduction

Embryo transfer (ET) is a critical step in assisted reproductive technology (ART) treatment. Frozen–thawed embryo transfer (FET) has become an effective and popular approach in ART mainly because of the development of vitrification [1]. FET cycles were associated with lower ovarian hyperstimulation syndrome risk and reduced risk of low birth weight, preterm birth, and small for gestational age infants [2, 3]. Among the endometrial preparation methods for FET, the hormone replacement therapy (HRT) protocol is quite popular because of its flexibility and convenience. However, endometrial receptivity may be impaired in the HRT cycle because of medication use [4].

Successful implantation requires good-quality embryos, receptive endometrium and synchronized embryo–endometrial crosstalk. The gonadotropin-releasing hormone (GnRH) pathway plays an important role in the hypothalamus-pituitary-gonadal axis of reproduction [5, 6]. Both GnRH and GnRH receptors (GnRH-R) are expressed not only in the hypothalamic pituitary but also in the endometrium and embryos; their expression reaches the highest levels at the secretory-phase endometrium and at the stage of expanded blastocyst [7–10]. Therefore, GnRH agonists (GnRHa) may have direct effects and functional roles in the endometrium and embryos. Indeed, GnRH has been reported to enhance endometrium receptivity and embryo development [11–14]. Several systematic reviews and meta-analyses indicated that adding GnRHa to progesterone significantly improved the ongoing pregnancy rate and live birth rate compared with using progesterone alone for luteal phase support in fresh embryo transfer (fET) cycles [15–18]. Furthermore, a systematic review and meta-analysis including 20 studies and 5497 patients demonstrated that GnRHa administration in the luteal phase boosted the clinical pregnancy rate in both fET and FET cycles, and the beneficial effect was similar between the fET and FET cycles [19].

GnRHa downregulation combined with HRT cycles has been increasingly used in recent years. Although the efficacy of GnRHa pretreatment is controversial [20, 21], a recent systematic review and meta-analysis of 27 articles with 14,152 patients reported that HRT cycles with GnRHa suppression were associated with an increased live birth rate and clinical pregnancy rate compared to those without GnRHa suppression [22]. Additionally, some studies have demonstrated that GnRHa pretreatment in HRT cycles may have a beneficial effect on specific groups, such as adenomyosis [23, 24], recurrent implantation failure (RIF) [25, 26] and thin endometrium [27]. We wondered whether additional administration of GnRHa during the luteal phase still takes effect in the GnRHa-HRT protocol. However, to date, no studies have investigated this issue. Thus, we designed this retrospective cohort study to assess the effects of the luteal-phase administration of single-dose GnRHa on reproductive outcomes in patients undergoing GnRHa-HRT FET cycles.

## Materials and methods

### Study design and participants

**Fig. 1:**
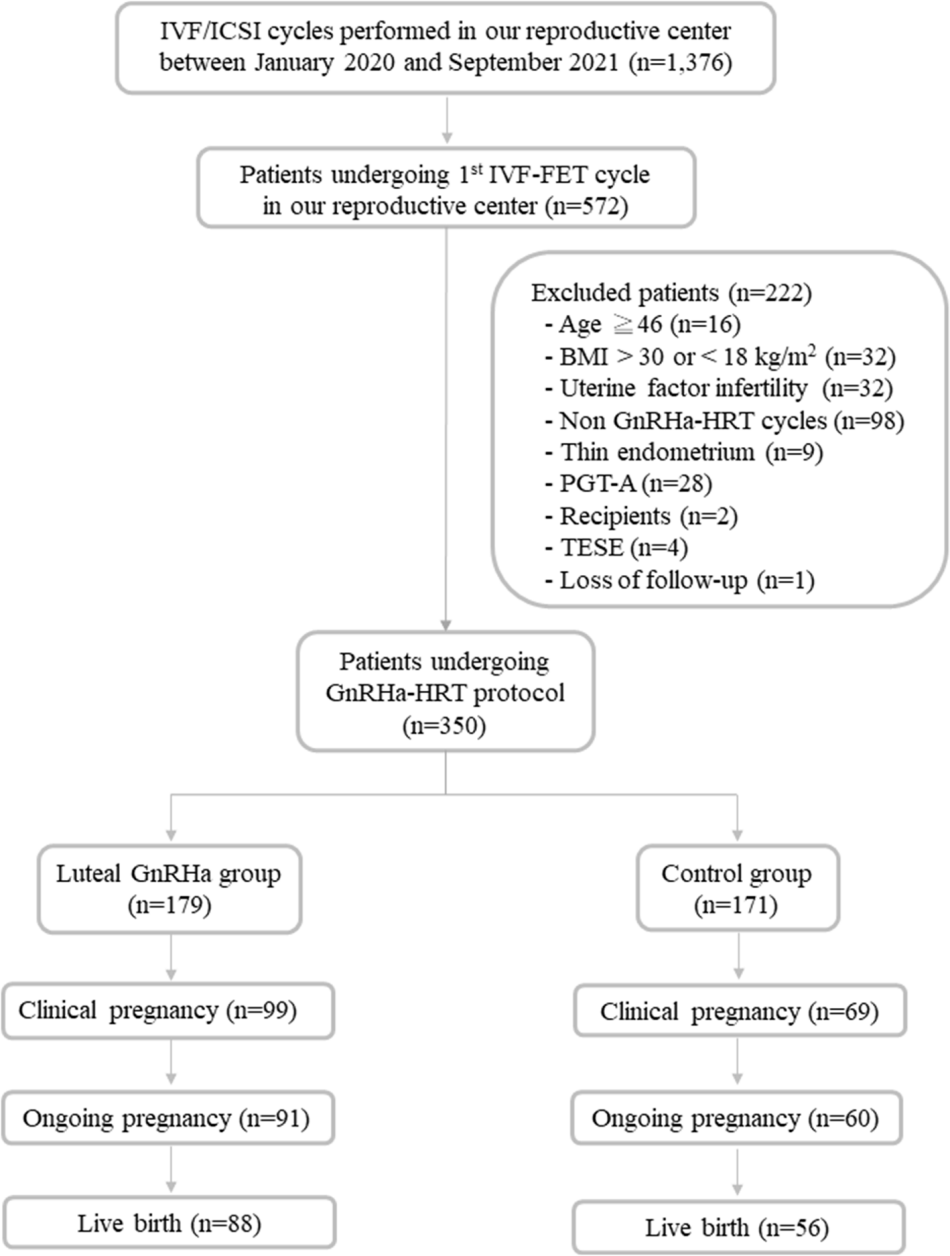
Study flow chart. IVF, in vitro fertilization; ICSI, intracytoplasmic sperm injection; FET, frozen–thawed embryo transfer; BMI, body mass index; GnRHa, gonadotropin-releasing hormone agonist; HRT, hormonal replacement therapy; PGT-A, preimplantation genetic testing for aneuploidy; TESE, testicular sperm extraction

This retrospective cohort study was performed at the Reproductive Medical Center of Kaohsiung Veterans General Hospital from January 2020 to September 2021. The study was approved by the Institutional Review Board of Kaohsiung Veterans General Hospital (reference number of institutional review board: KSVGH22-CT12-14). Because of its retrospective design, the requirement for consent was waived by the Institutional Review Board of Kaohsiung Veterans General Hospital. All patient data were collected from electronic medical records and in vitro fertilization (IVF) treatment sheets. Patients who received the first IVF-FET cycle in our reproductive medical center were included in this study. The exclusion criteria were as follows: (1) patients whose age was over 46 years old, (2) patients whose BMI was over 30 kg/m^2^ or less 18 kg/m^2^, (3) patients with uterine factor infertility, (4) patients who did not undergo GnRHa-HRT cycles, (5) patients who had thin endometrium (< 8 mm) after estradiol priming, (6) patients who received preimplantation genetic testing for aneuploidy (PGT-A), (7) patients who were oocyte recipients, (8) patients whose husbands underwent testicular sperm extraction (TESE) and (9) patients who were lost to follow-up. Finally, 350 patients undergoing the GnRHa-HRT protocol were identified and divided into the luteal GnRHa group (*n* = 179) and the control group (*n* = 171). The addition of luteal GnRHa administration was determined according to the patients’ consideration and preference after full consultation provided by a doctor. In the luteal GnRHa group, a single dose of GnRHa (Lupro 2 mg, Nang Kuang Pharmaceutical Co, Ltd., Tainan, Taiwan) was injected subcutaneously 2 hours after ET. The study flow chart is shown in Fig. 1.

### Endometrial preparation and frozen–thawed embryo transfer

All participants included in this study underwent FET cycles using the GnRHa-HRT protocol. A single injection of 3.75 mg long-acting GnRHa (Leuplin Depot, Takeda Pharmaceutical Company Limited, Yamaguchi, Japan) was given subcutaneously on menstruation cycle Day 2 or 3 after a thin endometrium (< 5 mm) was confirmed by transvaginal sonography. Twenty-eight to thirty days later, when the transvaginal sonography confirmed the presence of a thin endometrium (< 5 mm), endometrial preparation was commenced with daily oral estradiol 6-8 mg (Ediol, Synmosa Biopharma Corporation, Hsinchu County, Taiwan) and estradiol gel (Oestrogel gel, Besins, Drogenbos, Belgium). After consecutive administration for 14 days, a transvaginal ultrasound scan was performed to evaluate the endometrial thickness. If endometrial thickness was less than 8 mm, the dosage of estrogen was increased, and the medication duration was extended. If the endometrial thickness was still not sufficient after 20 days of estrogen administration, the cycle was cancelled. When the endometrial thickness reached at least 8 mm, luteal phase support was initiated using daily intravaginal gel 90 mg (Crinone 8% gel, Merck Serono, Hertfordshire, UK), daily oral dydrogesterone 30 mg (Duphaston, Abbott, Olst, the Netherlands) and intramuscular injection of progesterone 125 mg (Progeston Depot, Tafong Pharmaceutical Co., Ltd., Changhua City, Taiwan) twice a week.

A non-selective freeze-all strategy was introduced in our reproductive medical center. All embryos were cryopreserved using the vitrification technique. The cleavage-stage embryos and blastocysts were thawed and transferred on the 4th or 6th day after administration of progesterone, respectively. Day 3 embryo quality was evaluated according to the criteria from the Istanbul consensus workshop [28]. The percentage of fragmentation, the evenness of each blastomere and whether multinucleation was present were assessed to grade day 3 embryos as Grade 1 to Grade 3. Good-quality day 3 embryos were defined as 6-10 cells with Grade 2 (10-25% fragmentation, equal-sized blastomeres in the majority of cells and no multinucleation); top-quality Day 3 embryos were defined as 6-10 cells with Grade 1 (< 10% fragmentation, equal-sized blastomeres and no multinucleation) in this study. Day 5 embryo quality was assessed based on the Gardner and Schoolcraft scoring system. The degree of expansion (Grade 1-6), inner cell mass morphology (Grade A-C) and trophectoderm morphology (Grade A-C) were used to grade day 5 embryos. Good-quality day 5 embryos were defined as Grade 3BB; top-quality Day 3 embryos were defined as Grade 3AA in this study. ET was carried out under the guidance of transabdominal ultrasound. In the luteal GnRHa group, a single dose of GnRHa (Lupro 2 mg, Nang Kuang Pharmaceutical Co, Ltd., Tainan, Taiwan) was injected subcutaneously 2 hours after ET. Once pregnancy was achieved, luteal support was continued until 10–12 gestational weeks.

### Outcome measures

The primary outcome was live birth rate. The secondary outcomes included the clinical pregnancy rate, implantation rate and ongoing pregnancy rate. Biochemical pregnancy was confirmed by elevated serum β-human chorionic gonadotropin (hCG) levels (> 25 IU/L) at 14 days after ET. Clinical pregnancy was determined by visualization of fetal cardiac activity on transvaginal ultrasound at 6–7 weeks of gestation. Implantation rate was defined as number of intrauterine sac with a fetal heartbeat determined by transvaginal ultrasound by number of transferred embryos. A viable pregnancy beyond 12 weeks of gestation was considered ongoing pregnancy. Live birth was defined as the delivery of a viable fetus past 24 weeks of gestation. A loss of pregnancy before 24 weeks of gestation was regarded as miscarriage. Miscarriage was further divided into early miscarriage (≤ 12 weeks of gestation) and late miscarriage (> 12 weeks of gestation).

### Statistical analysis

The Kolmogorov–Smirnov test was used to test the normal distribution of continuous variables. Quantitative variables of normally distributed data, large enough samples, or both were assessed using Student’s t test; otherwise, Mann–Whitney U test was applied. Chi-squared test or Fisher’s exact test was used for comparing categorical data. Multivariable logistic regression was used to identify the independent effects of additional luteal GnRHa on live birth and ongoing pregnancy in all populations and patients with RIF after adjusting for age, body mass index, infertility duration, types of infertility, basal follicle-stimulating hormone (FSH), anti-Müllerian hormone (AMH), endometrial thickness, day of ET, number of transferred embryos and quality of transferred embryos. The results are shown as the odds ratio (OR) and 95% confidence interval (CI). A two-tailed value of *P* <  0.05 was considered statistically significant. Data processing and statistical analysis were carried out using IBM SPSS Statistics version 20.0 (IBM Corp., Armonk, NY, USA).

### Sample size calculation

The software G*Power 3.1 was used to calculate sample size. Live birth rate as the primary outcome was used to calculate sample size. Chi-squared test for independent samples was selected for the calculation. Live birth rate for the GnRHa-HRT protocol and HRT protocol were estimated to be 40 and 25%, respectively. With an alpha error of 0.05 and a power of 80%, it was postulated that the number of cases required for each group would be 152.

## Results

As shown in Fig. 1, a total of 1376 IVF/ICSI cycles were conducted from January 2020 until September 2021 in our reproductive medical center. During the period, 572 patients with their first IVF-FET cycle in our reproductive medical center were identified. Among the 572 patients, there were 16 patients who were older than 46 years old, 32 patients whose BMI was over 30 kg/m^2^ or less 18 kg/m^2^, 32 patients with uterine factor infertility, 98 patients who did not undergo GnRHa-HRT cycles, 9 patients who had thin endometrium (< 8 mm) after estradiol priming, 28 patients who received PGT-A, 2 patients who were oocyte recipients, 4 patients whose husbands underwent TESE, and 1 patient who was lost to follow-up. Those patients were excluded from the study. The remaining 350 patients with the GnRHa-HRT protocol were included and divided into the luteal GnRHa group (*n* = 179) and the control group (*n* = 171).

The baseline characteristics of the study population are summarized in Table 1. There were no significant differences between the two groups regarding age, body mass index, infertility duration, previous IVF attempts, infertility types and causes. Furthermore, basal FSH levels in the two groups were similar. However, lower AMH levels were found in the luteal GnRHa group than in the control group.

**Table 1:** Baseline characteristics of patients undergoing the GnRHa-HRT protocol with or without luteal GnRHa administration

Parameters	Luteal GnRHa group (*n* = 179)	Control group (*n* = 171)	*p* value
Age (years)	37.0 ± 4.2	37.1 ± 4.5	0.871
Body mass index (kg/m^2^)	23.0 ± 2.8	23.3 ± 2.9	0.465
Infertility duration (years)	4.4 ± 2.9	4.7 ± 2.9	0.332
Previous IVF attempts (%)			0.710
0-1	36.9%(66/179)	32.7%(56/171)	
2	22.9%(41/179)	25.1%(43/171)	
≧3	40.2%(72/179)	42.1%(72/171)	
Types of infertility (%)			0.541
Primary infertility	44.7%(80/179)	48.0%(82/171)	
Secondary infertility	55.3%(99/179)	52.0%(89/171)	
Causes of infertility (%)			0.708
Tubal factor	8.4%(15/179)	5.8%(10/171)	
Male factor	9.5%(17/179)	7.6%(13/171)	
POR	10.6%(19/179)	9.9%(17/171)	
PCOS	17.3%(31/179)	16.4%(28/171)	
Endometriosis	15.1%(27/179)	11.7%(20/171)	
Unexplained	12.8%(23/179)	15.8%(27/171)	
Multiple	26.3%(47/179)	32.7%(56/171)	
Basal FSH (IU/l)	4.7 ± 2.1	5.1 ± 4.2	0.249
Anti-Müllerian hormone(ng/mL)	3.39 ± 3.18	4.21 ± 3.90	0.036

Data are presented as the mean ± standard deviation or %

*GnRHa* gonadotropin-releasing hormone agonist, *HRT* hormonal replacement therapy, *IVF* in vitro fertilization, *POR* poor ovarian responders, *PCOS* polycystic ovarian syndrome, *FSH* follicle-stimulating hormone

As shown in Table 2, there were no significant differences between the two groups in terms of endometrial thickness, ET day, number of transferred embryos, percentage of ≥ one top-quality embryo transferred and quality of transferred embryos. A higher biochemical pregnancy rate (64.8% vs. 46.2%, *P* <  0.001), clinical pregnancy rate (55.3% vs. 40.4%, *P* = 0.005), implantation rate (33.4 ± 36.6% vs. 23.8 ± 33.6%, *P* = 0.012), ongoing pregnancy rate (50.8% vs. 35.1%, *P* = 0.003) and live birth rate (49.2% vs. 32.7%, *P* = 0.002) were observed in the luteal GnRHa group than in the control group. However, no significant difference in early and late miscarriage rate was observed between the two groups.

**Table 2:** Cycle characteristics of patients undergoing the GnRHa-HRT protocol with or without luteal GnRHa administration

Parameters	Luteal GnRHa group (*n* = 179)	Control group (*n* = 171)	*p* value
Endometrial thickness (mm)	11.1 ± 2.4	11.4 ± 2.6	0.164
Rate of ET day (%)			0.711
Day 3 ET	58.1% (104/179)	56.1% (96/171)	
Day 5 ET	41.9% (75/179)	43.9% (75/171)	
No. of transferred embryos	2.4 ± 0.8	2.5 ± 0.8	0.724
Quality of transferred embryos (%)			0.677
Good quality only	69.3% (124/179)	67.8% (116/171)	
Good and poor quality	27.4% (49/179)	26.9% (46/171)	
Poor quality only	3.4% (6/179)	5.3% (9/171)	
Rate of at least one top-quality embryo transferred (%)	83.8% (150/179)	83.6% (143/171)	0.965
Biochemical pregnancy rate (%)	64.8% (116/179)	46.2% (79/171)	< 0.001
Clinical pregnancy rate (%)	55.3% (99/179)	40.4% (69/171)	0.005
Implantation rate (%)	33.4 ± 36.6%	23.8 ± 33.6%	0.012
Ongoing pregnancy rate (%)	50.8% (91/179)	35.1% (60/171)	0.003
Live birth rate (%)	49.2% (88/179)	32.7% (56/171)	0.002
Miscarriage rate (%)	11.1% (11/99)	18.8% (13/69)	0.159
Early miscarriage rate (%)	8.1% (8/99)	13.0% (9/69)	0.294
Late miscarriage rate (%)	3.0% (3/99)	5.8% (4/69)	0.377

Data are presented as the mean ± standard deviation or %

*GnRHa* gonadotropin-releasing hormone agonist, *HRT* hormonal replacement therapy, *ET* embryo transfer

In Table 3, a binary logistic regression analysis was performed to assess the effects of additional luteal GnRH agonist in GnRHa-HRT cycles on ongoing pregnancy and live birth. Confounding parameters such as age, body mass index, infertility duration, types of infertility, basal FSH, AMH, endometrial thickness, day of ET, number of transferred embryos and quality of transferred embryos were included in the analysis. The multivariate analysis showed that the addition of a luteal GnRH agonist in GnRHa-HRT cycles had beneficial effects on the ongoing pregnancy rate (OR 2.04, 95% CI 1.20–3.47, *P* = 0.008) and live birth rate (OR 2.03, 95% CI 1.20–3.45, *P* = 0.009). Moreover, age and day of ET were independent factors that could affect the ongoing pregnancy rate and live birth rate.

**Table 3:** Analyses of factors affecting the ongoing pregnancy rate and live birth rate using logistic regression

	Ongoing pregnancy		Live birth	
	Adjusted OR^a^ (95% CI)	*p* value	Adjusted OR^a^ (95% CI)	*p* value
Luteal GnRHa vs. control	2.04 (1.20–3.47)	0.008	2.03 (1.20–3.45)	0.009
Age (years)	0.83 (0.76–0.90)	< 0.001	0.83 (0.76–0.90)	< 0.001
BMI (kg/m^2^)	1.09 (0.99–1.19)	0.078	1.07 (0.98–1.18)	0.145
Infertility duration (years)	1.04 (0.94–1.15)	0.421	1.02 (0.92–1.13)	0.676
Types of infertility	0.83 (0.48–1.43)	0.510	0.87 (0.50–1.49)	0.602
Basal FSH (IU/l)	0.94 (0.84–1.06)	0.307	0.91 (0.80–1.04)	0.161
AMH (ng/mL)	0.96 (0.88–1.05)	0.347	0.93 (0.85–1.02)	0.122
Endometrial thickness (mm)	1.03 (0.93–1.15)	0.519	1.00 (0.90–1.11)	0.959
Day of embryo transfer	2.32 (1.30–4.16)	0.005	2.32 (1.28–4.19)	0.005
No. of transferred embryos	1.03 (0.72–1.47)	0.878	1.07 (0.74–1.53)	0.724
Quality of transferred embryos	1.29 (0.77–2.16)	0.331	1.51 (0.89–2.56)	0.128

*OR* odds ratio, *CI* confidence interval, *BMI* body mass index, *FSH* follicle- stimulating hormone; *AMH* anti-Müllerian hormone

^a^Adjustment for age, BMI, infertility duration, types of infertility, basal FSH, AMH, endometrial thickness, day of embryo transfer, number of transferred embryos and quality of transferred embryos

We then attempted to investigate the effects of additional luteal GnRHa in patients with RIF. RIF was defined as failure to achieve a clinical pregnancy after at least three IVF or ICSI treatments with transfer of at least one good-quality embryo per transfer or transfer of 10 good-quality embryos according to a previous study [29]. As presented in Table 4, basal characteristics, including age, body mass index, infertility duration, infertility types, basal FSH and AMH, as well as cycle characteristics, including endometrial thickness, ET day, number of transferred embryos, percentage of ≥ one top-quality embryo transferred and quality of transferred embryos, were comparable between the two groups. Compared to the control group, the luteal GnRHa group had a significantly higher biochemical pregnancy rate (63.9% vs. 40.3%, *P* = 0.005), clinical pregnancy rate (54.2% vs. 30.6%, *P* = 0.004), ongoing pregnancy rate (47.2% vs. 23.6%, *P* = 0.004) and live birth rate (44.4% vs. 22.2%, *P* = 0.005). However, implantation rate and miscarriage rate were similar between the two groups. Furthermore, we performed a stratified analysis based on the day of embryo transfer and found that live birth rate was significantly higher in the luteal GnRHa group than in the control group both in the day 3 (38.5%% vs. 22.9%, *P* = 0.018) and day 5 (64.0%% vs. 45.3%, *P* = 0.022) embryo transfer (Supplementary Table 1).

**Table 4:** Subgroup analysis of RIF patients undergoing the GnRHa-HRT protocol with or without luteal GnRHa administration

Parameters	Luteal GnRHa group (*n* = 72)	Control group (n = 72)	*p* value
Age (years)	38.7 ± 3.9	38.9 ± 4.0	0.720
Body mass index (kg/m2)	23.1 ± 2.9	23.3 ± 3.1	0.616
Infertility duration (years)	5.7 ± 3.1	5.3 ± 3.2	0.334
Types of infertility (%)			0.165
Primary infertility	30.6% (22/72)	41.7% (30/72)	
Secondary infertility	69.4% (50/72)	58.3% (42/72)	
Basal FSH (IU/l)	4.7 ± 2.2	5.5 ± 6.0	0.335
Anti-Müllerian hormone (ng/mL)	2.67 ± 2.66	3.08 ± 3.41	0.426
Endometrial thickness (mm)	10.9 ± 2.3	11.1 ± 2.2	0.554
Rate of ET day (%)			0.113
Day 3 ET	72.2% (52/72)	59.7% (43/72)	
Day 5 ET	27.8% (20/72)	40.3% (29/72)	
No. of transferred embryos	2.7 ± 0.8	2.8 ± 0.9	0.922
Quality of transferred embryos (%)			0.302
Good quality only	62.5% (45/72)	54.2% (39/72)	
Good and poor quality	36.1% (26/72)	40.3% (29/72)	
Poor quality only	1.4% (1/72)	5.6% (4/72)	
Rate of at least one top-quality embryo transferred (%)	76.4% (55/72)	81.9% (59/72)	0.412
Biochemical pregnancy rate (%)	63.9% (46/72)	40.3% (29/72)	0.005
Clinical pregnancy rate (%)	54.2% (39/72)	30.6% (22/72)	0.004
Implantation rate (%)	25.8 ± 28.7	17.7 ± 31.3	0.108
Ongoing pregnancy rate (%)	47.2% (34/72)	23.6% (17/72)	0.003
Live birth rate (%)	44.4% (32/72)	22.2% (16/72)	0.005
Miscarriage rate (%)	17.9% (7/39)	27.3% (6/22)	0.393
Early miscarriage rate (%)	12.8% (5/39)	22.7% (5/22)	0.316
Late miscarriage rate (%)	5.1% (2/39)	4.5% (1/22)	0.919

Data are presented as the mean ± standard deviation or %

*RIF* recurrent implantation failure, *GnRHa* gonadotropin-releasing hormone agonist, *HRT* hormonal replacement therapy, *FSH* follicle-stimulating hormone, *ET* embryo transfer

As shown in Table 5, a binary logistic regression analysis was conducted to analyze the effects of additional luteal GnRHa in GnRHa-HRT cycles on ongoing pregnancy and live birth in the RIF subgroup. Age, body mass index, infertility duration, types of infertility, basal FSH, AMH, endometrial thickness, day of ET, number of transferred embryos and quality of transferred embryos were considered confounding factors in this analysis. The multivariate analysis revealed increased odds of ongoing pregnancy (OR 4.55, 95% CI 1.69–12.30, *P* = 0.003) and live birth (OR 4.30, 95% CI 1.59–11.65, *P* = 0.004) when luteal GnRHa was added in RIF patients undergoing the GnRHa-HRT protocol. In addition, age was negatively associated with ongoing pregnancy and live birth.

**Table 5:** Analyses of factors affecting the ongoing pregnancy rate and live birth rate in RIF patients using logistic regression

	Ongoing pregnancy		Live birth	
	Adjusted OR^a^ (95% CI)	*p* value	Adjusted OR^a^ (95% CI)	*p* value
Luteal GnRHa vs. control	4.55 (1.69–12.30)	0.003	4.30 (1.59–11.65)	0.004
Age (years)	0.85 (0.74–0.97)	0.014	0.85 (0.74–0.97)	0.018
BMI (kg/m^2^)	1.18 (0.99–1.41)	0.062	1.08 (0.91–1.28)	0.393
Infertility duration (years)	1.04 (0.87–1.24)	0.653	0.97 (0.81–1.17)	0.783
Types of infertility	0.62 (0.23–1.65)	0.333	0.67 (0.25–1.80)	0.426
Basal FSH (IU/l)	0.88 (0.70–1.10)	0.257	0.85 (0.67–1.08)	0.179
AMH (ng/mL)	1.00 (0.82–1.21)	0.988	0.97 (0.79–1.18)	0.734
Endometrial thickness (mm)	0.90 (0.73–1.10)	0.288	0.84 (0.67–1.04)	0.102
Day of embryo transfer	2.31 (0.79–6.80)	0.128	1.85 (0.63–5.43)	0.266
No. of transferred embryos	0.56 (0.29–1.07)	0.080	0.56 (0.29–1.08)	0.085
Quality of transferred embryos	0.54 (0.19–1.51)	0.237	0.47 (0.16–1.36)	0.164

*RIF* recurrent implantation failure, *OR* odds ratio, *CI* confidence interval, *BMI* body mass index, *FSH* follicle- stimulating hormone, *AMH* anti-Müllerian hormone

^a^Adjustment for age, BMI, infertility duration, types of infertility, basal FSH, AMH, endometrial thickness, day of embryo transfer, number of transferred embryos and quality of transferred embryos

## Discussion

This retrospective cohort study is the first to explore the possible effects of administration of single-dose GnRHa in luteal phase support on IVF outcomes in patients who underwent GnRHa-HRT cycles, and the results showed that additional luteal GnRHa was associated with a higher ongoing pregnancy rate and live birth rate. Moreover, the multivariate analysis revealed a 2.04-fold increase in the possibility of ongoing pregnancy (95% CI 1.20–3.47, *P* = 0.008) and a 2.03-fold increase in the possibility of live birth (95% CI 1.20–3.45, *P* = 0.009) when single-dose GnRHa was added to luteal phase support in patients receiving HRT cycles with GnRHa suppression. Luteal GnRHa administration potentially takes its effects in two ways: induction of endogenous pituitary gonadotropin release and in situ activation of GnRH-R in the endometrium and embryos. However, different from the HRT protocol, GnRHa-HRT protocol downregulates the pituitary and will inhibit pituitary gonadotropin release induced by luteal GnRHa administration. Therefore, luteal GnRHa supplementation in the GnRHa-HRT protocol only takes its effects through activation of the locally expressed GnRH/GnRH-R system in the endometrium and embryos.

GnRH analogs may have the potential to act on the endometrium and early stages of embryos. Both GnRH and GnRH-R have been reported to be expressed in both the epithelium and the stroma of the endometrium and reach the highest levels in the secretory phase of the menstrual cycle [7, 8, 30, 31], suggesting that the GnRH–GnRH-R pathway has functional roles in endometrium receptivity and implantation. GnRHa therapy may enhance the expression of endometrial integrin αvβ3, an adhesive molecule, and implantation-related factors, such as HOXA10 and LIF, in humans [11, 12] and mice [32, 33]. Furthermore, in a study of human decidual stromal cells, GnRH has been found to be able to modulate matrix metalloproteinases (MMPs) and their endogenous inhibitors, tissue-specific inhibitor of matrix metalloproteinases (TIMPs) [34], both of which are associated with cyclic remodeling of the endometrium and decidualization [35]. GnRHa has also been demonstrated to directly stimulate cell invasion and migration of human decidual endometrial stromal cells, a key process of embryo implantation and pregnancy programming [36]. These supported that GnRHa may facilitate endometrial receptivity and implantation. Moreover, both GnRH and GnRH-R have been shown to be present in human, mouse and porcine preimplantation embryos [9, 10, 13]. The levels of GnRH and GnRH-R significantly increased at the early blastocyst stage and reached their highest levels at the expanded blastocyst stage [10, 14]. In addition, the expression of GnRH and GnRH-R was detected in both the inner cell mass and trophectoderm cells of blastocysts [14]. These results implied that the GnRH–GnRH-R pathway could be a potential modulator of early embryo development. In animal models, treatment with GnRHa in culture medium can promote embryo development and inhibit apoptosis. In contrast, treatment with a GnRH antagonist suppressed embryo development and induced apoptosis via the intrinsic mitochondrial pathway, but the adverse effects could be reversed by cotreatment with GnRHa [10, 13, 14]. In addition, treatment with GnRH antagonist in mouse blastocysts significantly reduced the levels of EGF and IGF-II [14], which have been reported to be associated with embryo development and inhibition of apoptosis in blastocysts [37, 38]. In a study of human extravillous cytotrophoblasts, GnRHa was suggested to have the capacity to promote trophoblast invasion by regulating the expression of urokinase-type plasminogen activator (uPA) and plasminogen activator inhibitor (PAI-1) [39]. Additionally, GnRHa could regulate the synthesis and secretion of hCG in the preimplantation embryo and placenta [40, 41]. This suggests that GnRHa may be involved in early embryo development.

Taken together, GnRH analogs play an important role in the establishment of receptive endometrium and the development of peri-implantation embryos, by which GnRHa could facilitate successful implantation and pregnancy. However, more human studies are required to confirm the effects of GnRHa on the endometrium and embryos. A Cochrane meta-analysis including 10 randomized controlled trials (RCTs) and 2861 women demonstrated that live birth or ongoing pregnancy rates were higher in the progesterone plus GnRHa group than in the progesterone-only group [15]. Several other systematic reviews and meta-analyses reported similar results [16–18]. Furthermore, a systematic review and network meta-analysis of 89 RCTs with 29,625 women was performed to compare the effectiveness and safety of various methods of luteal phase support and showed that the addition of GnRHa to progesterone significantly improved the live birth rate compared to progesterone alone [42]. Another systematic review and network meta-analysis aimed to evaluate the effectiveness and safety of multiple-dose versus single-dose GnRHa protocols for luteal phase support in patients undergoing IVF/ICSI cycles. The results indicated that the multiple-dose GnRHa protocol might be the best strategy for improving the live birth rate and clinical pregnancy rate [43]. Nevertheless, these meta-analyses only included patients undergoing fET cycles. In terms of FET cycles, a systematic review and meta-analysis demonstrated that GnRHa administration in the luteal phase improved the clinical pregnancy rate in FET cycles, and the beneficial effect was similar to fresh cycles [19]. These studies supported our results in this article. However, a recent RCT including a total of 287 HRT–FET cycles showed no significant benefit in live birth rate and clinical pregnancy rate by administering two GnRHa boluses [44]. Therefore, more large-scale RCTs are needed to verify the beneficial effects of luteal GnRHa administration on IVF outcomes, especially in FET cycles.

Impaired endometrial receptivity and poor embryo quality were the two key causes of RIF [45]. The endometrium of the RIF patients showed decreased LIF expression and dysregulated LIF signaling [46, 47]. GnRHa therapy may boost the expression of implantation-related factors, such as HOXA10 and LIF [11, 12, 32, 33]. As mentioned above, culture medium with GnRHa can enhance embryo development and inhibit apoptosis [10, 13, 14]. Furthermore, immunological factor, such as altered Th1/Th2 ratio and imbalances of natural killer cells, might be another possible cause of RIF [45]. GnRHa was reported to play a direct role in immune modulation in patients with RIF and adenomyosis [48, 49]. Thus, we speculated that additional GnRHa administration during the luteal phase may have a beneficial effect on reproductive outcomes in women with RIF. The multivariate analysis of our study supported this speculation, showing a 4.55-fold increase in the possibility of ongoing pregnancy (95% CI 1.69–12.30, *P* = 0.003) and a 4.30-fold increase in the possibility of live birth (95% CI 1.59–11.65, *P* = 0.004) when luteal GnRHa was added to progesterone in RIF patients undergoing GnRHa-HRT cycles. However, we must interpret the data from the subgroup analysis discreetly because of the small population. More large-scale studies are required to prove our results.

One concern regarding GnRHa-HRT protocol is whether GnRH-R in the endometrium is also downregulated by long-acting GnRHa. Our data showed that luteal GnRHa administration still took effects in the GnRHa-HRT cycles. There might be four possibilities. First, GnRH-R in the endometrium was not downregulated by long-acting GnRHa. Second, long-acting GnRHa downregulated GnRH-R in the endometrium, but its effects faded out at the time of luteal GnRHa administration. Third, GnRH-R in the endometrium was downregulated by long-acting GnRHa. However, newly GnRH-R in the endometrium expressed during the luteal phase and was not downregulated by the long-acting GnRHa. Fourth, long-acting GnRHa downregulated GnRH-R in the endometrium and its effects continued during the luteal phase. The beneficial effects of luteal GnRHa administration came from acting on embryos. Further studies are required to investigate the issue. However, studies revealed that luteal GnRHa administration could improve fresh IVF outcomes under GnRHa long protocol [42]. Moreover, Xu et al. demonstrated that higher mRNA and protein expression of HOXA10, MEIS1 and LIF in endometrium were observed in the depot GnRHa protocol compared to the long GnRHa and GnRH antagonist protocols [12]. These studies seemed to support that GnRH-R in the endometrium was not downregulated by GnRHa. Nevertheless, more studies are needed to confirm the assumption.

This study has several limitations. First, the main limitation of this study was its retrospective design and limited sample size. Large-scale RCTs are needed to verify our results. Next, whether to administer luteal GnRHa was determined on the basis of patients’ consideration and preference after physician consultation, which may introduce bias. Patients may refuse to use it because of financial pressure. Moreover, embryo selection was based on morphological grading, not euploidy, because PGT-A has not been widely used in our center. Therefore, the confounding effects from embryo aneuploidy could not be excluded. Fourth, the data from the subgroup analysis should be interpreted cautiously on account of the potential bias from the small population.

In conclusion, our data suggest that single-dose administration of GnRHa during the luteal phase may improve the live birth rate in patients undergoing the GnRHa-HRT protocol.

## Supplementary Information

**Additional file 1: Supplementary Table 1.** Subgroup analysis (Day 3 or Day 5 embryo transfer) of patients undergoing the GnRHa-HRT protocol with or without luteal GnRHa administration.

## Data Availability

The datasets used and/or analyzed during the current study are available from the corresponding author on reasonable request.

## Abbreviations

AMH: Anti-Müllerian hormone
ART: Assisted reproductive technology
CI: Confidence interval
ET: Embryo transfer
fET: Fresh embryo transfer
FET: Frozen–thawed embryo transfer
FSH: Follicle-stimulating hormone
GnRH: Gonadotropin-releasing hormone
GnRHa: GnRH agonist
GnRH-R: GnRH receptor
HRT: Hormone replacement therapy
hCG: Human chorionic gonadotropin
ICSI: Intracytoplasmic sperm injection
IVF: in vitro fertilization
MMPs: Modulate matrix metalloproteinases
OR: Odds ratio
PAI-1: Plasminogen activator inhibitor
PGT-A: Preimplantation genetic testing for aneuploidy
RCTs: Randomized controlled trials
RIF: Recurrent implantation failure
TESE: Testicular sperm extraction
TIMPs: Tissue-specific inhibitor of matrix metalloproteinases
uPA: urokinase-type plasminogen activator
