# Supplementary material for: Additional single dose GnRH agonist during luteal phase support may improve live birth rate in GnRHa-HRT frozen–thawed embryo transfer cycle: a retrospective cohort study
Source: BMC Pregnancy Childbirth. 2023 Mar 14;23:174. doi: 10.1186/s12884-023-05491-y (PMC10012576; doi:10.1186/s12884-023-05491-y)
Supplement: Supplementary file 1 — Additional file 1: Supplementary Table 1. Subgroup analysis (Day 3 or Day 5 embryo transfer) of patients undergoing the GnRHa-HRT protocol with or without luteal GnRHa administration. [file 12884_2023_5491_MOESM1_ESM.doc]

**Supplementary Table 1 Subgroup analysis (Day 3 or Day 5 embryo transfer) of patients undergoing the GnRHa-HRT protocol with or without luteal GnRHa administration**

|  | Day 3 embryo transfer | | | Day 5 embryo transfer | | |
| --- | --- | --- | --- | --- | --- | --- |
| Parameters | Luteal GnRHa (n=104) | Control  (n=96) | *p* value | Luteal GnRHa (n=75) | Control  (n=75) | *p* value |
| Age (years) | 38.3±3.7 | 38.1±3.9 | 0.665 | 35.2±4.3 | 35.8±4.8 | 0.408 |
| Body mass index (kg/m2) | 22.8±2.9 | 23.2±2.9 | 0.328 | 23.3±2.7 | 23.3±3.0 | 0.954 |
| Infertility duration (years) | 4.6±3.1 | 4.4±2.8 | 0.567 | 4.0±2.6 | 5.0±3.0 | 0.028 |
| Types of infertility (%) |  |  | 0.051 |  |  | 0.189 |
| Primary infertility | 40.4% (42/104) | 54.2% (52/96) |  | 50.7% (38/75) | 40.0% (30/75) |  |
| Secondary infertility | 59.6% (62/104) | 45.8% (44/96) |  | 49.3% (37/75) | 60.0% (45/75) |  |
| Basal FSH (IU/l) | 4.7±2.3 | 5.2±2.3 | 0.128 | 4.8±1.9 | 5.1±6.0 | 0.699 |
| Anti-Müllerian hormone (ng/mL) | 2.40±2.83 | 3.02±3.17 | 0.153 | 4.74±3.13 | 5.81±4.24 | 0.089 |
| Endometrial thickness (mm) | 11.0±2.4 | 11.4±2.6 | 0.291 | 11.2±2.5 | 11.5±2.5 | 0.380 |
| No. of transferred embryos | 2.7±0.8 | 2.7±0.8 | 0.748 | 2.1±0.8 | 2.2±0.8 | 0.256 |
| Quality of transferred embryos (%) |  |  | 0.861 |  |  | 0.805 |
| Good quality only | 72.1% (75/104) | 45.8% (44/96) |  | 65.3% (49/75) | 64.0% (48/75) |  |
| Good and poor quality | 26.0% (27/104) | 45.8% (44/96) |  | 29.3% (22/75) | 28.0% (21/75) |  |
| Poor quality only | 1.9% (2/104) | 45.8% (44/96) |  | 5.3% (4/75) | 8.0% (6/75) |  |
| Rate of at least one top-quality embryos transferred (%) | 84.6% (88/104) | 83.3% (80/96) | 0.805 | 82.7% (62/75) | 84.0% (63/75) | 0.827 |
| Biochemical pregnancy rate (%) | 52.9% (55/104) | 33.3% (32/96) | 0.005 | 81.3% (61/75) | 62.7% (47/75) | 0.011 |
| Clinical pregnancy rate (%) | 45.2% (47/104) | 29.2% (28/96) | 0.019 | 69.3% (52/75) | 54.7% (41/75) | 0.064 |
| Implantation rate (%) | 21.4±28.0 | 14.4±25.3 | 0.065 | 49.9±40.7 | 35.9±38.8 | 0.032 |
| Ongoing pregnancy rate (%) | 39.4% (41/104) | 24.0% (23/96) | 0.019 | 66.7% (50/75) | 49.3% (37/75) | 0.032 |
| Live birth rate (%) | 38.5% (40/104) | 22.9% (22/96) | 0.018 | 64.0% (48/75) | 45.3% (34/75) | 0.022 |
| Miscarriage rate (%) | 14.9% (7/47) | 21.4% (6/28) | 0.470 | 7.7% (4/52) | 17.1% (7/41) | 0.164 |
| Early miscarriage rate (%) | 12.8% (6/47) | 17.9% (5/28) | 0.547 | 3.8% (2/52) | 9.8% (4/41) | 0.249 |
| Late miscarriage rate (%) | 2.1% (1/47) | 3.6% (1/28) | 0.707 | 3.8% (2/52) | 7.3% (3/41) | 0.461 |

Data are presented as the mean ± standard deviation and %.

GnRHa, gonadotropin-releasing hormone agonist; HRT, hormonal replacement therapy; FSH, follicle-stimulating hormone
